# A Novel Slope Method for Measurement of Fluid Density with a Micro-cantilever under Flexural and Torsional Vibrations

**DOI:** 10.3390/s16091471

**Published:** 2016-09-11

**Authors:** Libo Zhao, Yingjie Hu, Rahman Hebibul, Jianjun Ding, Tongdong Wang, Tingzhong Xu, Xixiang Liu, Yulong Zhao, Zhuangde Jiang

**Affiliations:** 1 State Key Laboratory for Manufacturing Systems Engineering, Collaborative Innovation Center of Suzhou Nano Science and Technology, Xi’an Jiaotong University, Xi’an 710049, China; hyj8880@foxmail.com (Y.H.); dianjianjun@126.com (J.D.); wtdwxy@163.com (T.W.); tingzhongxu@163.com (T.X.); leoxixiang@foxmail.com (X.L.); zhaoyulong@mail.xjtu.edu.cn (Y.Z.); zdjiang@mail.xjtu.edu.cn (Z.J.); 2 School of Automotive, Mechanical and Electrical Engineering, Xinjiang Vocational & Technical College of Communications, Urumqi 831401, China; rahman1963@163.com

**Keywords:** slope method, fluid density, micro-cantilever, nonresonant, free end excitation, double sided excitation

## Abstract

A novel method, which was called a slope method, has been proposed to measure fluid density by the micro-cantilever sensing chip. The theoretical formulas of the slope method were discussed and established when the micro-cantilever sensing chip was under flexural and torsional vibrations. The slope was calculated based on the fitted curve between the excitation and output voltages of sensing chip under the nonresonant status. This measuring method need not sweep frequency to find the accurate resonant frequency. Therefore, the fluid density was measured easily based on the calculated slope. In addition, the micro-cantilver was drived by double sided excitation and free end excitation to oscillate under flexural and torsional vibrations, respectively. The corresponding experiments were carried out to measure the fluid density by the slope method. The measurement results were also analyzed when the sensing chip was under flexural and torsional nonresonant vibrations separately. The measurement accuracies under these vibrations were all better than 1.5%, and the density measuring sensitivity under torsional nonresonant vibration was about two times higher than that under flexural nonresonant vibration.

## 1. Introduction

Vibration methods are widely used in online measurements of fluid density [1,2,3]. Micro Electromechanical Systems (MEMS) technology has increasingly been used to fabricate vibration sensors [4,5,6]. MEMS sensors need only a micro-scale fluid sample to measure its density, viscosity or other parameters. For instance, Corman [7] presented a fully low-pressure encapsulated and closed-loop operated resonant fluid density sensor, where the required sample volume was only 35 µL. A new simple silicon straight tube was tested as a fluid density sensor in the study of Najmzadeh [8]; the length of the proposed tube was 2.65 cm, and the volume of the fluid sample was only 9.3 µL. The micro-cantilever is one of most common structures used for MEMS vibration devices, as it is simple and reliable [9]. Wilson [10] showed that piezoelectric-excited millimeter-sized cantilever (PEMC) sensors could be used for determining both density and viscosity of liquids, and the measurement accuracy of fluid density was from 0.01% to 3.5%. Cakmak [11] introduced a novel method for fast measurements of liquid viscosity and density using two cantilevers with different geometries. The measurement error of the fluid density was better than 3% in the range of 995–1150 kg/m^3^. An edge-supported vibrating plate was fabricated using MEMS technology [12] to measure the density and viscosity of fluids. The density measurement error was better than 0.7%. However, all the sensors mentioned above were used to measure the fluid density by the resonant frequency shift under the flexural mode. The resonant modes of a micro-cantilever include not only flexural mode but also torsional [13] and lateral modes [14] and so on. In addition, the vibrating micro-cantilever must be immersed in the surrounding fluid, which results in an inertial load on the micro-cantilever, also called virtual mass [15]. Therefore, the change of fluid density contributes to the resonant frequency shift of micro-cantilever, however, its quality factor is not affected by the fluid density. 

At present, most MEMS vibrating sensors used to measure physical or biochemical quantities, work in resonance mode, such as when measuring fluid density via the resonant frequency shift [16,17]. Therefore, in order to guarantee the measurement accuracy, the resonant frequencies of the vibrating sensors must be accurately determined. In order to avoid calculating the resonant frequency, the novel slope method to measure the fluid density by the micro-cantilever sensing chip under the nonresonant working status was developed. The output voltage of a micro-cantilever sensor increases linearly with the increase of its driving voltage when the micro-cantilever is immersed in fluids of different densities. The different fluid density changes the vibration amplitude of the micro-cantilever. In addition, the output voltage of the Wheatstone bridge in the sensing chip is proportional to the stress of micro-cantilever sensing chip based on the piezoresistive principle, and its stress is proportional to its deflection, while its deflection is proportional to the driving voltage on the coil of the sensing chip. Then the slope is calculated from the curve representing the relationship between excitation and output voltages. It’s found that the slope is perfectly inversely proportional to the density of the surrounding fluid, therefore the fluid density can be measured from the corresponding slope. In addition, the fluid density measurement results of the slope method were also compared under two excitation modes: free end excitation and double sided excitation.

## 2. The Structure of the Sensing Chip and Two Different Excitation Ways

**Figure 1:**
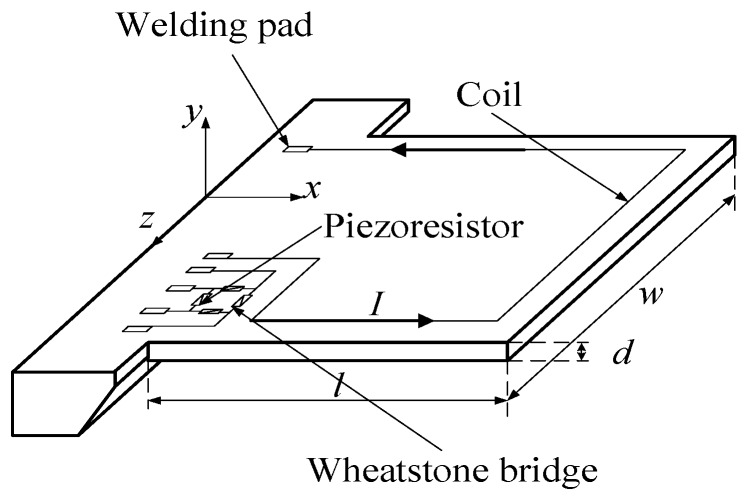
The structure of sensing chip with micro-cantilever.

In this paper, a sensing chip for the measurement of fluid density with a micro-cantilever structure was designed and fabricated from monocrystalline silicon using MEMS technology. Au was sputtered to fabricate the metallic coil on the micro-cantilever as shown in Figure 1. In the experiment, the sensing chip was put in a uniform magnetic field and the alternating voltage at a certain frequency, also called driving voltage, powered the coil. Then, a Lorentz force was generated to drive the micro-cantilever vibrating at the same frequency of the alternating voltage. For the vibrating micro-cantilever, the stress would concentrate on its fixed end where four piezoresistors were fabricated by a doping process to constitute a Wheatstone bridge. Therefore, the vibrating signal of the micro-cantilever could be detected by the output voltage of Wheatstone bridge based on the piezoresistive principle. Welding pads were used to bond Au wires for the connection with external circuits.

In Figure 1, *l*, *w* and *d* denote the length, width and thickness of the micro-cantilever, respectively, where *l* = 1500 μm, *w* = 2500 μm and *d* = 30 μm. The micro-cantilever has different vibrating modes under different excitations, such as flexural, torsional and lateral vibrations. This research was just concentrated on the flexural and torsional vibrations, which were excited in two different ways.

**Figure 2:**
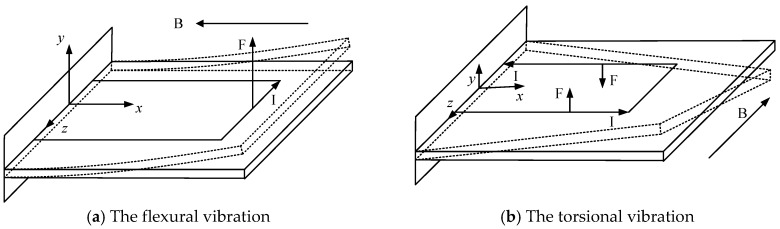
Two different vibrating forms under different excitation ways.

As shown in Figure 2a, the Lorentz force was generated and applied to the free end of micro-cantilever when the direction of magnetic field was parallel to the *x* axis. The flexural vibration of the micro-cantilever was generated for the sensing chip under this condition. This driving way is called free-end excitation in this research.

The torsional vibration of the micro-cantilever was excited by a torque as shown in Figure 2b. The Lorentz force was generated and applied to the double long sides of the micro-cantilever when the direction of the magnetic field was parallel to the *z* axis. This driving mode is called double sided excitation in this research.

## 3. The Slope Method Under Nonresonant Vibration

**Figure 3:**
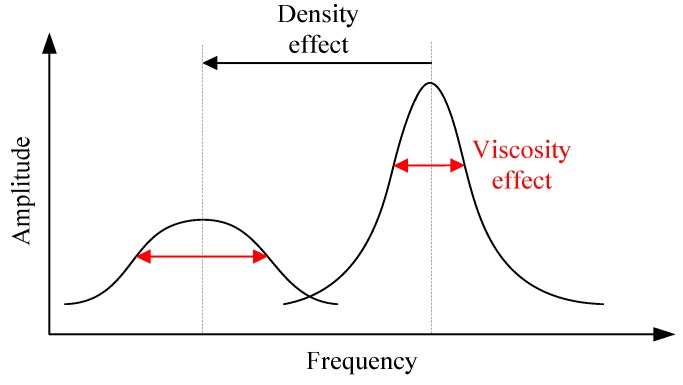
The density and viscosity effects under resonance.

As is known to all, the resonant frequency of a vibrating device immersed in a fluid will be influenced mainly by the fluid density, and the full width at half maximum (FWHM) of the device will be affected by the fluid viscosity [18], as shown in Figure 3. The Reynolds number *Re* of an oscillating cantilever is 2*πfρw*^2^/(4*η*), where *η* and *ρ* are viscosity and density of the fluid, *w* is the width and *f* is the resonant frequency of the cantilever. For high *Re* (*Re* >> 1), the viscous effect is negligible and the resonance frequency of the cantilever is primarily a function of fluid density and independent of fluid viscosity [19].

In this paper, the width of the proposed micro-cantilever was 2500 μm, and the *Re* was about 100 and far larger than 1. Therefore, the viscosity effect can be neglected for this sensing chip when the fluid viscosity is not very large (≤10 mPa·s). The experimental results in Section 3.2 also prove this statement. When we researched the density effect on the vibration of the micro-cantilever under its nonresonant status, the fluid density could be treated as a virtual mass applied to the micro-cantilever. The effects of fluid density on the micro-cantilever are discussed in detail as follows. 

### 3.1. Under Flexural Nonresonant Vibration

**Figure 4:**
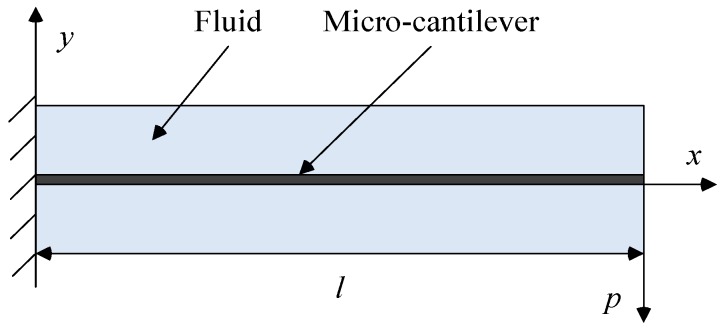
The cross section of the micro-cantilever in a fluid.

The cross section of the rectangular micro-cantilever in the fluid is shown in Figure 4. The Lorentz force is generated as a simple harmonic force *p*(*l*) and applied to the free end of the micro-cantilever. The deflection amplitude in the free end of micro-cantilever is directly proportional to the force *p*(*l*) because monocrystalline silicon is a linear elastic material.

Without considering the effect of fluid viscosity, the differential equation of the micro-cantilever under flexural vibration in the fluid can be written as [19]:

(1) $$\frac{\partial^{2}}{\partial x^{2}}\left(EJ\frac{\partial^{2}y}{\partial x^{2}}\right)+\rho_{e}A\frac{\partial^{2}y}{\partial t^{2}}=p(l)\sin\omega t$$

where *E* is the Young modulus, *J* is moment of inertia, *ρ*_e_ is the equivalent density of the micro-cantilever in the fluid, *A* is the cross sectional area of the micro-cantilever. Because the cross section of the rectangular micro-cantilever is constant, Equation (1) can be expressed as:

(2) $$\frac{\partial^{4}y}{\partial x^{4}}+\frac{1}{a^{2}}\frac{\partial^{2}y}{\partial t^{2}}=\frac{1}{EJ}p(l)\sin\omega t$$

where:

(3) $$a^{2}=\frac{EJ}{\rho_{e}A}$$

The deflection of the stationary response on the free end of the micro-cantilever was presumed as:

(4) y(l,t)=w(l)sinωt

where *w*(*l*) is the maximum deflection of micro-cantilever along *y* direction.

After Equation (4) was substituted into Equation (2):

(5) $$w^{\left(4\right)}(x)-\beta^{4}w(x)=\frac{1}{EJ}p(l)$$

where:

(6) $$\beta^{4}=\frac{\omega^{2}}{a^{2}}$$

After solving Equation (5), the general solution of the deflection on the free end of the micro-cantilever can be presented as follows:

(7) $$w(l)=C_{1}\cos\beta l+C_{2}\sin\beta l+C_{3}\cosh\beta l+C_{4}\sinh\beta l+C_{5}\frac{p(l)}{2EJ\beta^{4}}-C_{6}$$

where *C*_i_ (i = 1~6) is constant.

When the vibration force on the free end of a micro-cantilever changes, the vibration amplitude is also changed correspondingly. When the excitation force is defined as *p*_1_(*l*), the deflection amplitude of the free end of micro-cantilever is denoted by *w*_1_(*l*), and when the excitation force is *p*_2_(*l*), the deflection amplitude of its free end is changed to *w*_2_(*l*), and the following equation is obtained based on Equation (7):

(8) $$\frac{w_{2}(l)-w_{1}(l)}{p_{2}(l)-p_{1}(l)}=\frac{C_{5}}{2\rho_{e}A\omega^{2}}$$

where:

(9) $$\rho_{e}=\rho_{s}+C_{7}\rho_{f}$$

In Equation (9), *ρ_s_* is the density of micro-cantilever and *ρ_f_* is the density of fluid, *C*_7_ is a constant.

An output voltage *U* of the Wheatstone bridge, which is located on the fixed end of the micro-cantilever, has a linear relationship with the deflection amplitude of the free end of the micro-cantilever [20]. Furthermore, the excitation force applied to the micro-cantilever also has a linear relationship with the driving voltage *V* on the coil. 

Therefore:

(10) w(l)∝U

(11) p(l)∝V

After substituting Equations (9)–(11) into Equation (8), we obtain the following function:

(12) $$\frac{U_{2}(l)-U_{1}(l)}{V_{2}(l)-V_{1}(l)}=\frac{C_{5}}{2(\rho_{s}+C_{7}\rho_{f})A\omega^{2}}$$

### 3.2. Under Torsional Nonresonant Vibration

**Figure 5:**
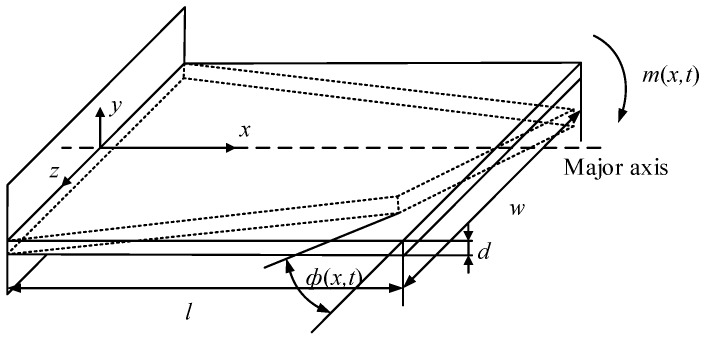
Sketch of the micro-cantilever under torsional vibration.

The torsional vibration sketch of the micro-cantilever was shown in Figure 5. When a simple harmonic torque *m*(*x,t*) is applied to the micro-cantilever, it vibrates torsionally along the major axis (*x*) to generate a deflection angle *ϕ*(*x*,*t*).

The vibration differential equation of the micro-cantilever in the fluid under torsional vibration could be written as [21]:

(13) $$GK\frac{\partial^{2}\phi (x,t)}{\partial x^{2}}-\rho_{e}I\frac{\partial^{2}\phi (x,t)}{\partial t^{2}}=m(x,t)\sin\omega t$$

where *G* is the shear modulus of the micro-cantilever, and *I* is the polar moment of inertia about the major axis of rotation. The parameter *K* in Equation (13) is a torsional constant related to the cross-sectional area. For a rectangular cross section with width *w* and thickness *d*, *K* is given by [22]:

(14) $$K=\frac{1}{3}d^{4}\left(\frac{w}{d}-0.63\right)$$

Equation (14) is an approximation based on the assumption that the cross section is a narrow rectangle with *w* > *d*. Equation (13) could be solved like Equation (1) in Section 3.1. The relationship between the output voltage *U* of the Wheatstone bridge and the driving voltage *V* on the coil under torsional vibration can be expressed as:

(15) $$\frac{U_{2}(l)-U_{1}(l)}{V_{2}(l)-V_{1}(l)}=\frac{D_{1}}{(\rho_{s}+C_{7}\rho_{f})ID_{2}}$$

where *D*_1_ and *D*_2_ are also constants.

Equations (12) and (15) have a similar form; their left parts are actually the slopes (denoted by *k*) of the fitted curves that are obtained from the corresponding output voltages of the Wheatstone bridge and different driving voltages on the coil. 

Therefore, both Equations (12) and (15) can be simplified to demonstrate the relationship between the fluid density and slope *k* as:

(16) $$\rho_{f}=\frac{m}{k}+n$$

where *m*, *n* are constants and can also be calculated by experimental calibration. The fluid density can then be calculated from the slope *k* using Equation (16), which is called the slope method.

## 4. Experiments

**Figure 6:**
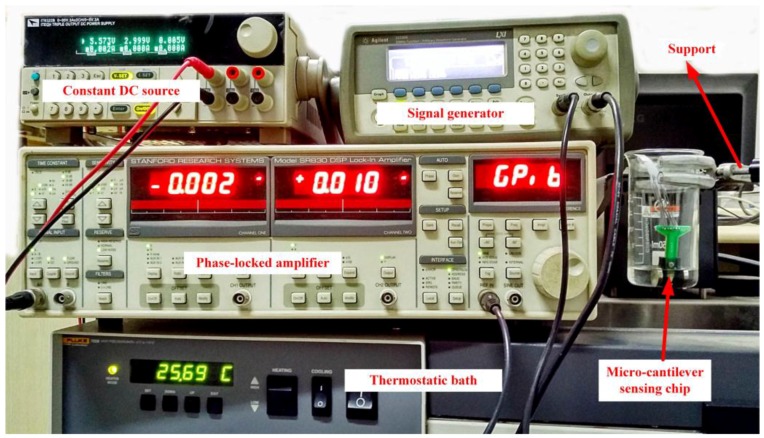
The experimental system.

An experimental system had been established as shown in Figure 6. The micro-cantilever sensing chip was immersed in the fluid to be measured, and the vessel filled with the fluid was fixed with a support and could drop into a thermostatic bath Fluke 7008 (Fluke, Everett, WA, USA). 

A SR830 lock-in amplifier (Stanford Instruments, Sunnyvale, CA, USA) detected the output voltage *U* of the Wheatstone bridge when the sinusoidal AC voltage as the driving voltage *V* excited the metal coil at a constant frequency. The driving voltage *V* was provided by a 33220 A signal generator (Agilent, Santa Clara, CA, USA) and a constant current of 2 mA was provided by an ITECH IT6233B DC source (ITECH, Nanjing, China) to power the Wheatstone bridge.

### 4.1. The Two Different Excitations 

**Figure 7:**
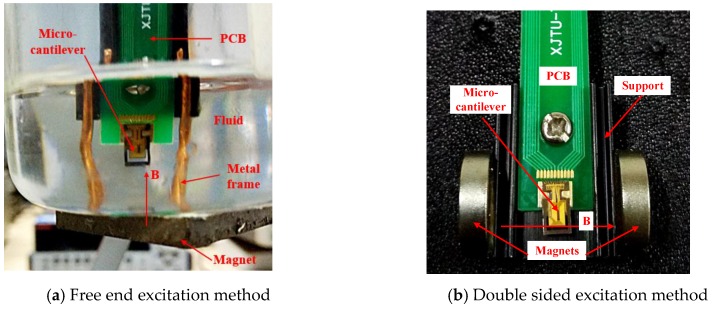
Schematics of different excitation methods.

The free end excitation method is explained in Figure 7a. The micro-cantilever sensing chip was fixed on a Printed circuit board (PCB). The PCB was fixed on a support that could dip into the fluid. A magnet was pasted on the bottom of vessel to provide a stationary magnetic field. The micro-cantilever must be close to the surface of the magnet, which ensures the magnetic induction lines around the micro-cantilever are uniform and parallel to the *x* axis of the micro-cantilever. A metal frame around the micro-cantilever as shown in Figure 7a was used to protect the sensing chip. The double sided excitation method was demonstrated in Figure 7b. There were two magnets pasted on two sides of a support with the width of 20 mm to provide a magnetic field. Then, the magnetic induction lines were uniform and parallel to *z* axis of the micro-cantilever.

### 4.2. The Influencing Parameters for the Slope Method

**Figure 8:**
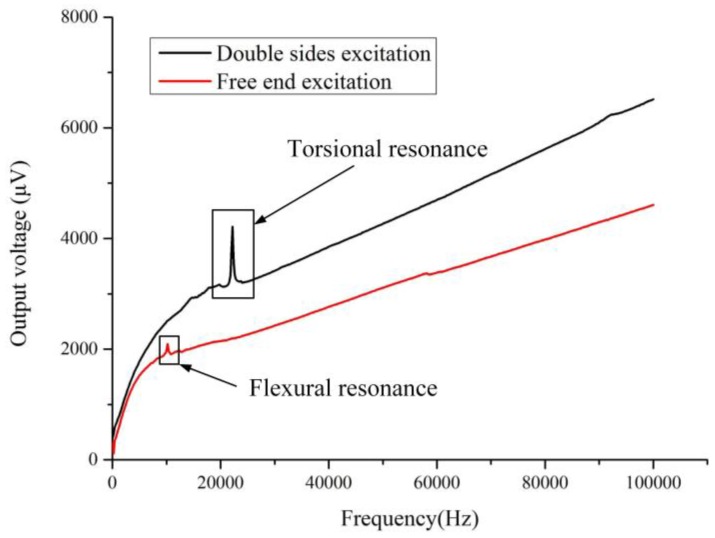
The output voltage of the Wheatstone bridge versus sweeping frequency under the two excitation methods.

The experimental parameters of the two excitation modes are discussed in this section. First, the driving voltage *V* on the coil should not be larger than 1000 mV to ensure the vibration amplitude of the micro-cantilever is far smaller than any dimension of the sensing chip. Figure 8 shows the output voltage *U* of the Wheatstone bridge under the driving voltage *V* with 800 mV in sweeping frequency from 10 Hz to 100 kHz with steps of 200 Hz. The interval time between two adjacent testing points was one second. The measuring process was completed in octane at 30 °C. In Figure 8, for the sweeping curve under free end excitation, the resonant peak was the 1st flexural resonance, and the 2nd order resonance did not appear as its amplitude was too weak. For the sweeping curve under double sided excitation, the resonant peak was the 1st torsional resonance, and the flexural resonance could not be excited. The fluid density changed the resonant frequency of the micro-cantilever, so the relationship between the slope and the fluid density was not linear when the vibrating frequency of the micro-cantilever was around its resonant frequency. The frequency of the proposed micro-cantilever for the slope method was selected larger than 30 kHz and away from its resonant frequency. In addition, the vibrating frequency should be lower to reduce energy consumption. Summing up the above, the vibrating frequency of the micro-cantilever in the experiments of Section 4 was fixed at 50 kHz.

The effect of viscosity on the measurement of the fluid density by the slope method was researched in the next experiment. In order to evaluate the effect of viscosity, silicone oil was selected as the sample. The viscosity values of silicone oils range from 0.5 mPa·s to 100 mPa·s, but their density value changes are very small, only from 0.93 kg·m^−3^ to 0.97 kg·m^−3^. Based on Equations (12) and (15), the values of slope *k* were calculated after the micro-cantilever sensing chip was immersed in the selected silicone oils under different excitation modes. 

**Figure 9:**
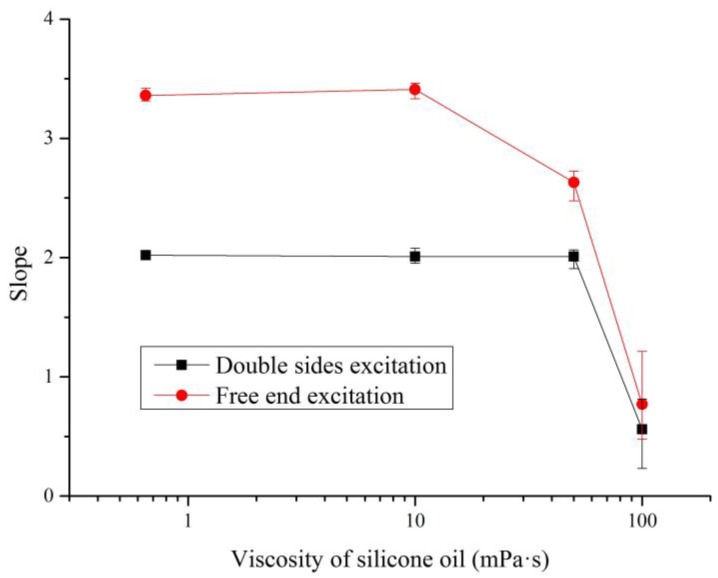
The effect of viscosity on the slope.

As shown in Figure 9, the values of slope *k* were all about 2 when the viscosity values of silicone oils were 0.65 mPa·s, 10 mPa·s and 50 mPa·s (the black curve) under double sided excitation, but it became 0.56 when the viscosity value was 100 mPa·s. However, in the red curve corresponding to free end excitation, the values of slope *k* were all about 3.4 when the viscosity values of silicone oils were 0.65 mPa·s and 10 mPa·s, but they became 2.63 and 0.77 when the viscosity values were 50 mPa·s and 100 mPa·s, respectively. The experimental results illustrate that the slope method for the measurement of fluid density was not significantly affected by the viscosity when the viscosity value of measured fluid was less than 10 mPa·s. Therefore, a low viscosity value can be neglected for the proposed slope method to measure the density of a fluid.

## 5. Results and Discussion of the Slope Method 

The experiments for the density measurements of five fluids were carried out under different temperatures of 25 °C, 30 °C and 35 °C, the corresponding reference values of their densities are shown in Table 1. The reference values of density, viscosity and sonic speed under standard atmospheric pressure and different temperature were calculated using the Reference Fluid Properties (REFPROP) software. This program, developed by the U.S. National Institute of Standards and Technology (NIST), calculates the thermodynamic and transport properties of industrially important fluids and their mixtures. A small temperature range had been chosen in these experiments in order to avoid the temperature influence on the piezoresistors. In addition, the alkane fluids easily volatilize at temperatures beyond 35 °C.

### 5.1. Measuring Results of Fluid Density Under Flexural Nonresonant Vibration

**Figure 10:**
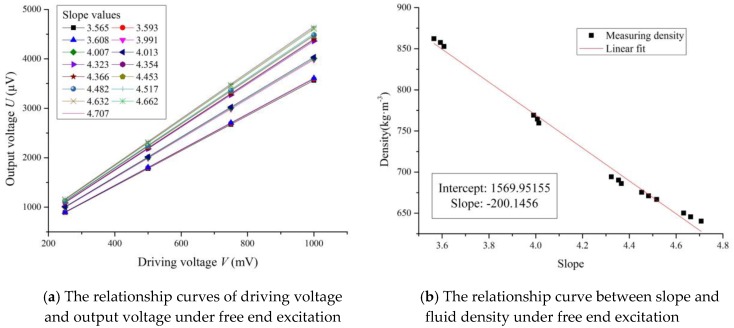
The measuring curves under free end excitation.

The sensing chip was packaged as shown in Figure 7a, and the micro-cantilever was driven to be vibrating under free end excitation. The driving voltage *V* on the coil was from 250 mV to 1000 mV with steps of 250 mV. There were 15 different density values of five fluids under three different temperatures. The output voltage *U* of the Wheatstone bridge was tested 10 times at every measuring point, and the final output voltage *U* of the Wheatstone bridge at every measuring point was the average value of these measurement results. As shown in Figure 10a, each curve was obtained and fitted to show the relationship of the driving voltage *V* and output voltage *U* when the micro-cantilever sensing chip was immersed in each fluid under each temperature. Also, the corresponding slope values for the fitted curves were calculated and are shown in Figure 10a.

The reference density values of *n*-hexane and methylbenzene under 30 °C were used to calibrate the working Equation (16). Thus, the constants of *m* and *n* were calculated as:

*m* = 3249.986, *n* = −46.963

Then, the working Equation (16) became:

(17) $$\rho_{f}=\frac{3249.986}{k}-46.963$$

Therefore, the density values of the fluids at different temperatures measured by the slope method under free end excitation were calculated using Equation (17) and are shown in Table 2. The absolute relative errors between the measured results and reference values of different fluid densities were all less than 1.1%.

As shown in Figure 10b, the relationship curve between the slope *k* and fluid density under free end excitation was fitted from the data in Table 2. The slope k decreased linearly with the increasing fluid density, which was consistent with Equation (17). The absolute average measuring accuracy was about 0.46%. In addition, the density measurement sensitivity (DMS) under free end excitation could be expressed as S = Δk/Δ*ρ_f_* = 0.005 (kg·m^−3^)^−1^, where the unit of slope *k* was 1.

### 5.2. Measuring Results of Fluid Density Under Torsional Nonresonant Vibration

**Figure 11:**
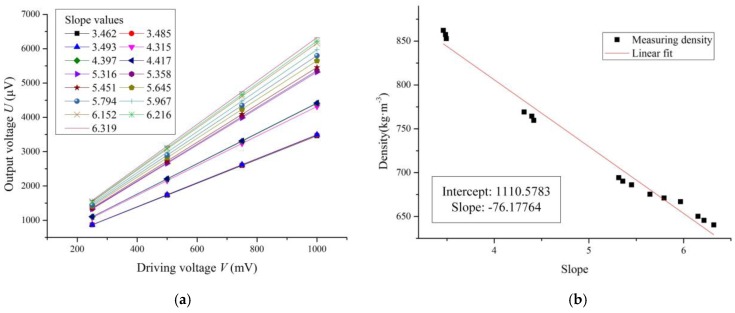
The measuring curves under double sided excitation. (**a**) The relationship curves of driving voltage and output voltage under double sided excitation; (**b**) The relationship curve between slope and fluid density under double sided excitation.

The sensing chip was packaged as shown in Figure 7b, and the micro-cantilever was driven to be vibrating by double sided excitation. The other conditions in these measurement experiments were all same as in the free end excitation experiments. Each curve was obtained and fitted as in Figure 11a to show the relationship between the driving voltage *V* and output voltage *U* when the micro-cantilever sensing chip was immersed in each fluid at each temperature. In addition, the corresponding slope values were also calculated and are shown in Figure 11a.

The reference density values of *n*-hexane and methylbenzene under 30 °C were also used to calibrate the working Equation (16). Thus, the constants of *m* and *n* were calculated as:

*m* = 1645.212 and *n* = 385.486

Then, the working Equation (16) was changed to:

(18) $$\rho_{f}=\frac{1645.212}{k}+385.486$$

The other density values of fluids by the slope method under double sided excitation were calculated using Equation (18) and are shown in Table 3. The absolute relative errors between the measured results and reference values of the different fluid densities were all less than 1.5%.

As shown in Figure 11b, we could obtain the fitted curve between slope *k* and fluid density under double sided excitation from the data in Table 3. The slope *k* also decreased linearly with increasing fluid density, which is consistent with Equation (18). The absolute average measuring accuracy was about 0.60%. In addition, the DMS under double sided excitation was S = Δ*k*/Δ*ρ_f_* = 0.013 (kg·m^−3^)^−1^, which was above two times that under free end excitation.

It can be seen from Figure 10a and Figure 11a that the output voltage of the sensing chip under double sided excitation was larger than that under free end excitation. The main reason was the stress concentration of micro-cantilever under double sided excitation was greater than that under free end excitation. Therefore, the output voltage of the Wheatstone bridge was larger under double sided excitation based on the piezoresistive principle, which made the slope increase, so the DMS was larger under double sided excitation, but the fluid density measuring accuracies were almost same under both types of excitation according to the experimental results.

## 6. Conclusions

A novel slope method for the measurement of fluid density was presented. The slope between driving and output voltages of a sensing chip under nonresonant status was inversely proportional to the density of the surrounding fluid. This measurement method did not need to find accurate resonance frequencies via the sweeping frequency in a large frequency range. The relationship between fluid density and slope has been deduced under flexural and torsional nonresonant vibrations. The influences of frequency and viscosity on the measurement of fluid density by the slope method were studied in experiments. The slope method could be used to measure fluid density with good accuracy when the viscosity value of the measured fluid was less than 10 mPa·s. The fluid density measurement accuracies in the range from 640 kg·m^−3^ to 870 kg·m^−3^ were 0.46% and 0.60% by the slope method under free end excitation and double sided excitation, respectively. The experimental results showed that the measurement accuracy under double sided excitation was not better than under free end excitation, but the DMS of 0.013 (kg·m^−3^)^−1^ under double sided excitation was better than that under free end excitation (0.005 (kg·m^−3^)^−1^). Therefore, it’s better for micro-cantilever sensing chip to measure fluid density under torsional vibration by the slope method. 

The slope method significantly shortens the measuring time of fluid density compared with traditional measurement methods via the resonant frequency. It just needs to obtain two groups of voltage points to calculate the slope. Accordingly, the resonant method needs to sweep the frequency to obtain the resonant frequency, and it was hard to calculate the accurate resonant frequency over a large range, thus the slope method greatly improves the fluid density measurement efficiency.

Nevertheless some disadvantages still exist in this study. For example, the sensor cannot work in conductive liquids because there was no isolation layer to protect the coil and pads, so a future research direction will be to design a new chip with a protective layer to measure the density of conductive liquidd, and some experiments will be carried out in the fluids with densities larger than 1000 kg/m^3^. In addition, the noise investigation in the measurement is another important research topic for future study.

## Figures and Tables

**Table 1:** Reference density and viscosity values of different fluids.

	Temperature/°C	*n*-Hexane	*n*-Heptane	Octane	Cyclohexane	Methylbenzene
Density/kg·m^−3^	25	654.78	679.60	698.27	773.89	862.24
	30	650.16	675.36	694.24	769.14	857.57
	35	645.51	671.10	690.20	764.37	852.89
Viscosity/mPa·s	25	0.2963	0.3885	0.5097	0.8847	0.5526
	30	0.2815	0.3675	0.4805	0.8163	0.4926
	35	0.2677	0.3482	0.4538	0.7553	0.4664

**Table 2:** The density measurement results by the slope method under free end excitation.

Fluid	*T*/°C	*k*	*ρ_f_*/kg·m^−3^	Error/%
*n*-Hexane	35	4.707	643.49532	−0.312106711
	30	4.662	650.1599727	0
	25	4.632	654.6750159	−0.016033494
*n*-Heptane	35	4.517	672.5382741	0.214315914
	30	4.482	678.1568672	0.414129821
	25	4.453	682.8791819	0.482516462
Octane	35	4.366	697.4225268	1.046439692
	30	4.354	699.4741167	0.753934766
	25	4.323	704.8267749	0.939002807
Cyclohexane	35	4.013	762.9017156	−0.192090787
	30	4.007	764.1143901	−0.65340639
	25	3.991	767.3660146	−0.843011977
Methylbenzene	35	3.608	853.8094452	0.107803496
	30	3.593	857.5699746	0
	25	3.565	864.6742986	0.282322624

**Table 3:** The density measurement results by the slope method under double sidedexcitation.

Fluid	*T*/°C	*k*	*ρ_f_*/kg·m^−3^	Error/%
*n*-Hexane	35	6.319	645.8458031	0.052021361
	30	6.216	650.1599969	0
	25	6.152	652.9134294	−0.285068361
*n*-Heptane	35	5.967	661.2047012	−1.474489464
	30	5.794	669.437232	−0.876979389
	25	5.645	676.9321282	−0.392565014
Octane	35	5.451	687.3046269	−0.419497695
	30	5.358	692.5433549	−0.244388847
	25	5.316	694.9693135	−0.472694868
Cyclohexane	35	4.417	757.9589511	−0.838736329
	30	4.397	759.6531638	−1.233434255
	25	4.315	766.7636349	−0.920849878
Methylbenzene	35	3.493	856.4887907	0.421952502
	30	3.485	857.5700016	0
	25	3.462	860.7063173	−0.1778719

## References

[1] Gruszkiewicz M.S., Rother G., Wesolowski D.J., Cole D.R., Wallacher D. (2012). Direct measurements of pore fluid density by vibrating tube densimetry. Langmuir.

[2] Comuñas M.J.P., Bazile J.P., Baylaucq A., Boned C. (2008). Density of diethyl adipate using a new vibrating tube densimeter from (293.15 to 403.15) K and up to 140 MPa. Calibration and measurements. J. Chem Eng. Data.

[3] Sanmamed Y.A., González-Salgado D., Troncoso J., Cerdeiriña C.A., Romaní L. (2007). Viscosity-induced errors in the density determination of room temperature ionic liquids using vibrating tube densitometry. Fluid Phase Equilib..

[4] Khan M.F., Schmid S., Larsen P.E., Davis Z.J., Yan W., Stenby E.H., Boisen A. (2013). Online measurement of mass density and viscosity of pL fluid samples with suspended microchannel resonator. Sens. Actuators B Chem..

[5] Burg T.P., Manalis S.R. (2003). Suspended microchannel resonators for biomolecular detection. Appl. Phys. Lett..

[6] Doy N., McHale G., Newton M.I., Hardacre C., Ge R., Allen R.W., Maclnnes J.M. Separate density and viscosity determination of room temperature ionic liquids using dual quartz crystal microbalances. Proceedings of the 2009 IEEE Sensors Conference.

[7] Corman T., Enoksson P. (2000). A low-pressure encapsulated resonant fluid density sensor with feedback control electronics. Meas. Sci. Technol..

[8] Najmzadeh M., Haasl S., Enoksson P. (2007). A silicon straight tube fluid density sensor. J. Micromech. Microeng..

[9] Saha S., Topkar A., Rathod S.S. Study of MEMS microcantilever based on their geometric parameters. Proceedings of the 2014 International Conference on Advanced Communication Control and Computing Technologies.

[10] Wilson T.L., Campbell G.A., Mutharasan R. (2007). Viscosity and density values from excitation level response of piezoelectric-excited cantilever sensors. Sens. Actuators A Phys..

[11] Cakmak O., Ermek E., Kilinc N., Yaralioglu G.G., Urey H. (2015). Precision density and viscosity measurement using two cantilevers with different widths. Sens. Actuators A Phys..

[12] Goodwin A.R.H. (2008). A MEMS vibrating edge supported plate for the simultaneous measurement of density and viscosity: Results for Argon, Nitrogen, and Methane at temperatures from (297 to 373) K and pressures between (1 and 62) MPa. J. Chem. Eng. Data.

[13] Cai T., Josse F., Heinrich S., Nigro N., Dufour I., Brand O. Resonant characteristics of rectangular microcantilevers vibrating torsionally in viscous liquid media. Proceedings of the 2012 IEEE International Conference on Frequency Control Symposium.

[14] Cox R., Zhang J., Josse F., Heinrich S., Dufour I., Beardslee L.A., Brand O. Damping and mass sensitivity of laterally vibrating resonant microcantilevers in viscous liquid media. Proceedings of the 2012 IEEE International Joint Conference on Frequency Control and the European Frequency and Time Forum.

[15] Ghatkesar M.K., Rakhmatullina E., Lang H.P., Gerber C., Hegner M., Braun T. (2008). Multi-parameter microcantilever sensor for comprehensive characterization of Newtonian fluids. Sens. Actuators B Chem..

[16] Lucklum F., Reichel E.K., Jakoby B. (2011). Miniature density–viscosity measurement cell utilizing electrodynamic-acoustic resonator sensors. Sens. Actuators A: Phys..

[17] Lederer T., Stehrer B.P., Bauer S., Jakoby B., Hilber W. (2011). Utilizing a high fundamental frequency quartz crystal resonator as a biosensor in a digital microfluidic platform. Sens. Actuators A Phys..

[18] Hur D., Lee J.H. (2013). Determination of liquid density and viscosity using a self-actuating microcantilever. Jpn. J. Appl. Phys..

[19] Sader J.E. (1998). Frequency response of cantilever beams immersed in viscous fluids with applications to the atomic force microscope. J. Appl. Phys..

[20] Zhao L., Xu L., Zhang G., Jiang Z., Zhao Y., Wang J.H., Wang X.P., Liu Z.G. (2014). In-situ measurement of fluid density rapidly using a vibrating piezoresistive microcantilever sensor without resonance occurring. IEEE Sens. J..

[21] Turner J.A., Wiehn J.S. (2001). Sensitivity of flexural and torsional vibration modes of atomic force microscope cantilevers to surface stiffness variations. Nanotechnology.

[22] Timoshenko S.P., Goodier J.N. (1987). Theory of Elasticity.

